# Validity of a New 3-D Motion Analysis Tool for the Assessment of Knee, Hip and Spine Joint Angles during the Single Leg Squat

**DOI:** 10.3390/s20164539

**Published:** 2020-08-13

**Authors:** Igor Tak, Willem-Paul Wiertz, Maarten Barendrecht, Rob Langhout

**Affiliations:** 1 Physiotherapy Utrecht Oost, Bloemstraat 65D, 3581 WD Utrecht, The Netherlands; 2 Amsterdam Collaboration on Health and Safety in Sports, Amsterdam UMC, Vrije Universiteit Amsterdam, Department of Public and Occupational Health, Amsterdam Public Health Research Institute, Van der Boechorststraat 7, 1081 BT Amsterdam, The Netherlands; maartenbarendrecht@hotmail.com; 3 Knowledge Centre of Sports and Physical Activity, 6717 LZ Ede(GLD) Horapark 4, The Netherlands; willem-paul.wiertz@kenniscentrumsportenbewegen.nl; 4 Dutch Center for Allied Health Care (NPi), Berkenweg 7, 3818 LA Amersfoort, The Netherlands; rob.langhout@fysiotherapiedukenburg.nl; 5 Avans+, Heerbaan 14-40, 4817 NL Breda, The Netherlands; 6 Physiotherapy Dukenburg Nijmegen, Aldenhof 70-03, 6537 DZ Nijmegen, The Netherlands

**Keywords:** validity, 3-D motion analysis, single leg squat, motion capture, clinical

## Abstract

Aim: Study concurrent validity of a new sensor-based 3D motion capture (MoCap) tool to register knee, hip and spine joint angles during the single leg squat. Design: Cross-sectional. Setting: University laboratory. Participants: Forty-four physically active (Tegner ≥ 5) subjects (age 22.8 (±3.3)) Main outcome measures: Sagittal and frontal plane trunk, hip and knee angles at peak knee flexion. The sensor-based system consisted of 4 active (triaxial accelerometric, gyroscopic and geomagnetic) sensors wirelessly connected with an iPad. A conventional passive tracking 3D MoCap (OptiTrack) system served as gold standard. Results: All sagittal plane measurement correlations observed were very strong for the knee and hip (*r* = 0.929–0.988, *p* < 0.001). For sagittal plane spine assessment, the correlations were moderate (*r* = 0.708–0.728, *p* < 0.001). Frontal plane measurement correlations were moderate in size for the hip (ρ = 0.646–0.818, *p* < 0.001) and spine (ρ = 0.613–0.827, *p* < 0.001). Conclusions: The 3-D MoCap tool has good to excellent criterion validity for sagittal and frontal plane angles occurring in the knee, hip and spine during the single leg squat. This allows bringing this type of easily accessible MoCap technology outside laboratory settings.

## 1. Introduction

The evaluation of athletes’ kinematics in functional, sports-specific situations continues to receive increasing attention [1,2]. Kinematic parameters like the range of motion (ROM), velocity and acceleration are used to quantify so-called quality of movement [1]. Quality of movement is associated with injury risk in athletes, and is evaluated in clinical practice to determine exercise progression and assist in return to play decision making after injury [1,3,4]. In sports involving running, cutting and jumping, decreased spine, hip and knee flexion have been linked to the development of patellofemoral pain and increased strain of the anterior cruciate ligament (ACL) [5,6,7].

Three-dimensional (3D) motion capture (MoCap) systems using reflective markers are considered the gold standard in measuring kinematics during functional performance tests [8,9]. However, feasibility and financial considerations have forced clinicians to adopt two-dimensional (2D) rather than 3D analysis for commonly used functional performance tests, such as the single leg squat [10]. Even though it is considered a useful screening tool [9,11,12,13,14], most kinematic 2D analyses of the single leg squat still require the use of multiple markers and devices like cameras and computers and its use is still time consuming [3,9,14]. Like the single leg squat, most performance tests are executed in a fixed position like single leg stance, and are thus easily reproducible. Movement quality is then often judged visually, thereby lacking quantitative data. A huge step forward in movement quality assessment would then be to track 3D movement outside the laboratory and in clinical settings with easy-to-use, low cost and time efficient systems [4]. Obtaining larger data sets is then made possible by the multi-site use of this technology [2].

Registration of 3D kinematics allows for a more comprehensive assessment of compensatory movement patterns, incorporating all anatomical planes, thus resulting in a smaller loss of relevant data [1,8,9,12,13,15]. Smart devices (tablets and phones) are now commonly equipped with cameras, Bluetooth connectivity and data processing capacity. Wearable active sensors can communicate with these devices, making 3D MoCap easily accessible for clinicians. A previous review on the reliability of motion capture systems reported the highest reliability for hip and knee sagittal and frontal plane measurements while it is lowest for the transverse plane measurements [16]. Reliability of wearable active sensor systems is moderate to excellent when compared to optical tracking systems with sagittal and frontal hip and knee measurement error between sagittal 8° and 1° [4,17,18]. Active sensors recently proved to be useful and reliable in the assessment of squatting, jumping and walking in patients after ACL reconstruction [19]. Currently, active sensor monitoring of movement is the subject of many studies in the field of orthopedic and neurologic rehabilitation and sports injury populations [17,20,21,22].

This new field of rapidly emerging technologic possibilities however needs further evaluation before it can be applied in the field by researchers and clinicians [23]. The aim of this study was to determine the concurrent validity of a new 3D MoCap tool for sagittal and frontal plane angles of the knee, hip and spine during a single leg squat.

## 2. Materials and Methods

A cross-sectional study design was used. The single leg squat task was analysed using both conventional 3D motion analysis and a newly developed 3D MoCap tool. At the time of this study this product was not yet available on the market. All procedures were carried out at the same time of the day.

### 2.1. Subjects

Subjects were recruited from the local student population at the campus of De Haagse Hogeschool (The Hague, The Netherlands). Subjects were included if they were aged between 18 and 45 years and were physically active (activity level ≥5/10 on the Tegner activity scale [24] for at least of 60 min per week).

Exclusion criteria were: (1) a history of knee surgery; (2) knee injuries or back, hip, knee or ankle joint pain within 3 months prior to this study; (3) lower extremity symptoms at rest or during sports participation; and (4) any neurological disorders that could affect gait. This study was conducted according the Helsinki declaration of ethical principles for medical research involving human subjects [25]. The Dutch Central Committee on Research Involving Human Subjects (CCMO) [26] confirmed exemption from ethical approval as stated in the Dutch Medical Research Involving Human Subjects Act [27]. All subjects signed informed consent before participating in this study.

### 2.2. Motion Capture

The primary outcome measures of this study were derived from conventional 3D motion analysis and from a new 3D MoCap tool during a repeated single leg squat test.

An inert 3D motion analysis system consisting of 12 infrared cameras (OptiTrack Flex 13, NaturalPoint Inc., Corvallis, OR, USA) was used as a reference tool. The positions of reflective markers in 3D space (x, y and z axes), were recorded by the infrared cameras at a frequency of 120 Hz. Although radiostereometric analysis (RSA) is considered the gold standard in motion analysis, a major disadvantage is its invasive character. RSA has highest accuracy, yet comparison with optical tracking systems shows clinical acceptable validity and reliability [28,29]. Systems like Vicon (Vicon, Oxford, UK) and OptiTrack are therefore commonly used in motion analysis [3,8,9]. OptiTrack systems were found to be accurate when compared to Vicon in gait assessment with the deviations reported being up to 2.2% maximum [30,31]. Hip and knee ROM data in the sagittal and frontal plane were reported to differ 1° maximum [30].

The new 3D MoCap tool (SportsLapp, Factic BV, Enschede, The Netherlands [32]) used 4 active (triaxial accelerometric, gyroscopic and geomagnetic) sensors (Hocoma AG, Volketswil, Switzerland), attached with elastic therapeutic tape (Pinotape Pro Sport, Pino Pharmaceutical Products GmbH, Hamburg, Germany). The sensors connected wirelessly by Bluetooth, to an iPad Air 2 (Apple Inc., Cupertino, CA, USA) with the SportsLapp software running on the iOS (Apple Inc., Cupertino, CA, USA) platform. Joint angles were registered using the sensors (recording at 50 Hz) and video (recording at 120 Hz). The lower sensor data capturing frequency was graphically aligned in the movement curves with the video recording frequency by spherical linear interpolation. Data collected during one single set of 3 trials was used for analyses. This protocol is similar to other protocols reported in literature [13,15,33,34].

### 2.3. Testing Protocol

**Figure 1:**
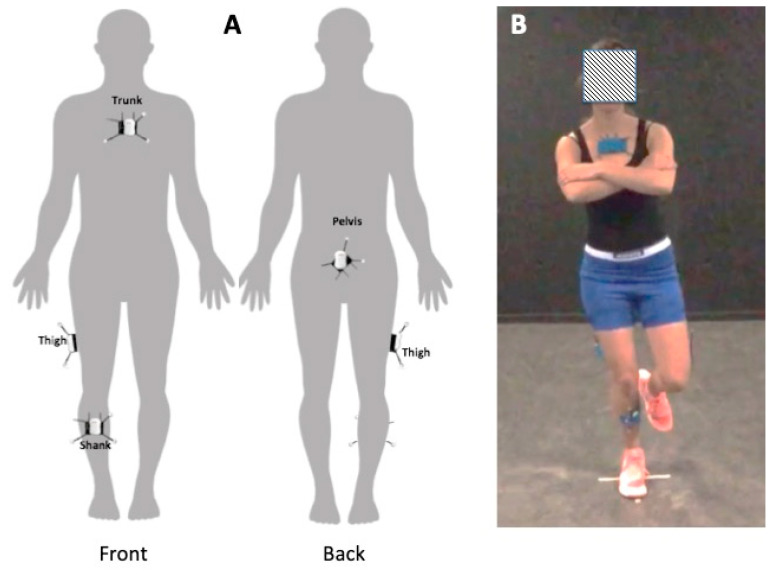
(**A**): The ventral view (left) and dorsal view (right) show the cluster marker and sensor positions on the sternal manubrium, the sacrum, halfway on the iliotibial tract and halfway on the tibia. (**B**): The single leg testing position with markers and sensors attached.

For the conventional motion analysis and the 3D MoCap tool, cluster markers and active sensors respectively, were placed on the following anatomical landmarks: the sternal manubrium, the sacrum, halfway on the lateral aspect of the thigh and halfway, anteromedial on the shank (bony aspect of the tibia), see Figure 1.

Prior to testing, subjects performed a standardised warming up consisting of 8 bipedal squats and 3 single leg squats on each leg. Then both systems were calibrated with the subjects standing in the upright anatomical position, while all joint angle values on both systems were set to be zero.

For the single leg squat test, the protocol as described by Dingenen et al. [3] was used. Subjects had to stand on their dominant leg (defined as the preferred leg to kick a ball) and fold their arms in front of their chest. They then squatted down in 2 s and returned to the upright position in 2 s, while maintaining balance. This low movement speed was selected to minimize the chance for trajectory gaps [31]. The non-supporting knee had to be kept parallel to the supporting knee, without touching it. During the test, squat depth was not controlled, as this better reflects a clinical setting. It was reported previously that subjects are able to produce consistent sagittal plane range of motion without monitoring [35,36]. In order to reduce the effect of movement velocity on joint angles and lower limb kinematics, a metronome was used to provide an audio cue for speed of movement [3,13,34].

### 2.4. Data Processing

All data from the conventional 3D motion analysis system were imported into MatLab R2015b (The MathWorks Inc., Natick, MA, USA), and marker trajectories were filtered using a 4th order low-pass Butterworth filter at 3 Hz, eliminating all signal noise with a frequency higher than 3 Hz. A custom MatLab program—including a Euler rotation sequence resolving the sagittal, the frontal and the transverse plane motion respectively—was used to calculate angles of the spine, hip and knee. Euler angles of rotation describe complex 3D kinematics of a rigid body (i.e., a limb segment) by decomposing movements into rotations about the 3 axes (x, y and z) of a fixed coordinate system that serves as a reference [37]. Spine flexion and homolateral spine lateral flexion, and hip and knee flexion, adduction and internal rotation were considered positive values.

**Figure 2:**
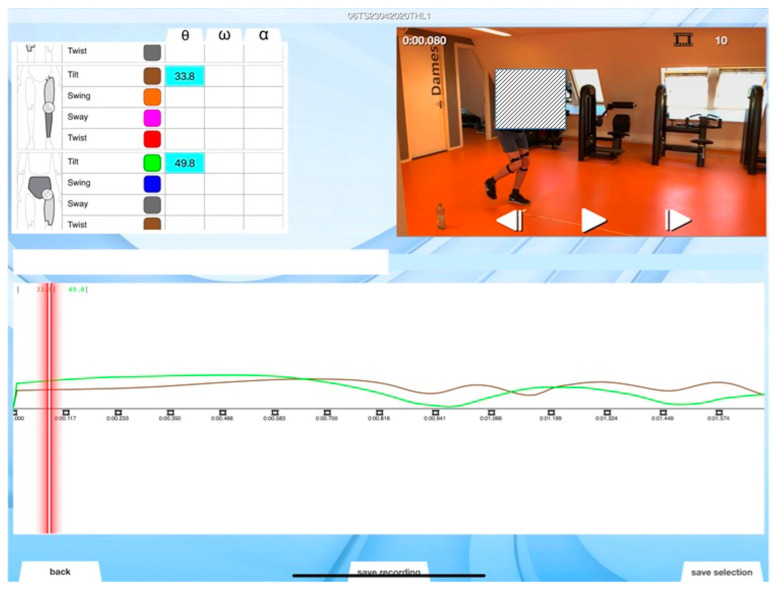
Screenshot of the SportsLapp app, with on-screen representation of video and kinematic data. Here left hip and knee data are visible.

For the new 3D MoCap tool, custom-made software (SportsLapp, Factic BV, Enschede, The Netherlands) was used, analysing the collected kinematic data and converting it into real-time joint angle curves. Both the curves and the video footage are available simultaneously in the app (see Figure 2).

Each of the sensors was assigned to a specific limb segment. As a joint consists of 2 segments, joint angles were defined as the angular difference between 2 segments. From each sensor, a quaternion was derived, expressing the orientation and rotation of its specific segment in the local coordinate frame of a primary segment (generally the proximal adjacent segment). Quaternions are a way of mathematically encoding the 3D orientation of a limb segment using 4 scalar numbers; 3 representing vectors on the axes of rotation, and 1 providing the angle of rotation [38]. In this way, they are a means of overcoming gimbal lock—a singular joint position in which 2 of the 3 axes of rotation align, and thus joint angles and kinematics cannot be described accurately [39]. The additional dimension in a quaternion provides more information on the orientation of a segment so that gimbal lock can be avoided.

**Figure 3:**
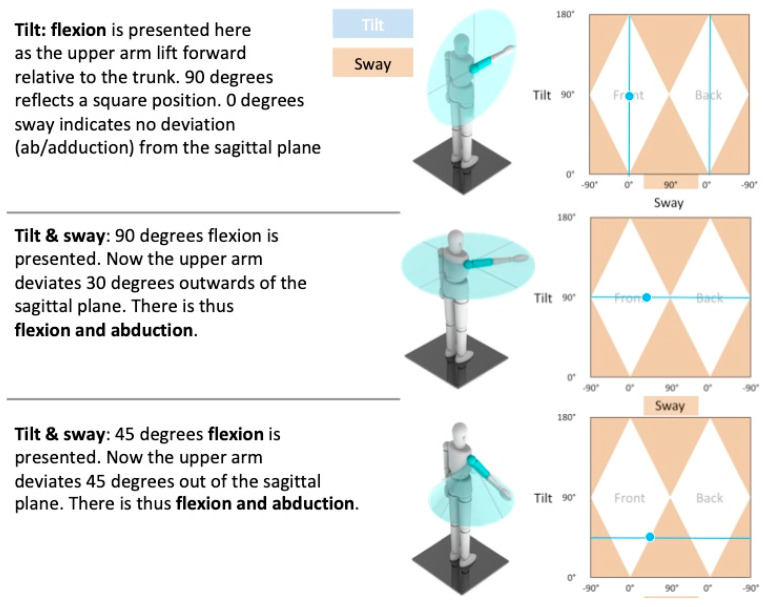
A graphical representation of sagittal and frontal plane movement of the SportsLapp app.

For the new 3D MoCap tool, active sensors register joint angles. There is no calibration against fixed x, y, and z-axes in 3D space. Thus all joint positions are based on data obtained from 2 adjacent segments, relatively positioned against one another. To describe these 3D joint angles and movements, the 3D MoCap tool employs a new kinematic system (3D Angles, Factic BV, Enschede, The Netherlands) [40] consisting of 4 units of measurement: tilt, swing, sway and twist presented in the SportsLapp software. The amount of tilt effectively quantifies the amount of movement of a (secondary) segment with respect to its primary segment. Thus when performed in the sagittal plane, tilt describes flexion and extension of the spine, hip and knee. By analogy, sway shows movement in lateral and in medial direction. So when performed in the frontal plane, sway describes spine lateral flexion and hip abduction and adduction. Twist quantifies rotation along the longitudinal axis of the segment (transversal plane). For this study we applied sagittal plane (tilt) and frontal plane (sway) analyses (see Figure 3).

### 2.5. Outcome Measures

The kinematic variables collected were: knee flexion (tilt), hip flexion (tilt) and abduction/adduction (sway), spine forward flexion (tilt) and lateral flexion angles (sway). These were all presented in degrees, rounded off to 1 decimal. Both 3D motion analysis systems registered the entire movement trajectory during the single leg squat performed. In order to reduce eventual effects of velocity on joint angles registered, kinematic data at the time point of peak knee flexion were collected for analysis by the following procedure: The SportsLapp software creates a graphical curve of the joint angles. Moreover, the software allows selecting a certain part of the curve (depicting the moment where peak knee flexion position occurred) and then provides the start time and end time of the interval. By searching the raw IMU data corresponding to this interval, the magnitude of peak knee flexion and the time point at which this occurred was determined.

### 2.6. Statistical Analysis

In order to determine characteristics of our sample, mean, standard deviation and range were calculated for the subjects’ age, height, weight, hours of sport participation per week and activity level. To investigate the concurrent validity of the new MoCap tool, correlations between outcomes on both 3D motion analysis systems were calculated. To test for normality of data distribution, Shapiro-Wilk tests were conducted. In case of normally distributed data, Pearson’s *r* was calculated—in case of non-normally distributed data, Spearman’s Rho (ρ) was used. Strength of correlations were expresses as perfect (*r* or ρ = −1 or 1), very strong (0.8 ≤ *r* to ρ < 1), moderate (0.6 ≤ *r* to ρ < 0.8), fair (0.3 ≤ *r* to ρ < 0.6) and poor (0 < *r* to ρ < 0.3) or inversely the negative values in case of negative correlations [41]. The alfa level for statistical significance was set at *p* < 0.05 for all analyses. Statistical analyses were performed using IBM SPSS Statistics (v. 23.0, IBM Corp., Armonk, NY, USA).

## 3. Results

### 3.1. Subjects

Forty-four subjects (24 males and 20 females) took part in the experiment. Subject characteristics are presented in Table 1.

### 3.2. Concurrent Validity

All single leg squats were performed 3 times resulting in a total of 132 trials being available for analyses. The data recorded per trial for both systems is presented in Table 2 as mean, standard deviation (SD) and 95% confidence interval (CI). Pearson’s R (normally distributed data) or Spearman’s Rho (non-normally distributed data) was calculated between data obtained with both systems including the mean differences with accompanying 95% CI.

**Figure 4:**
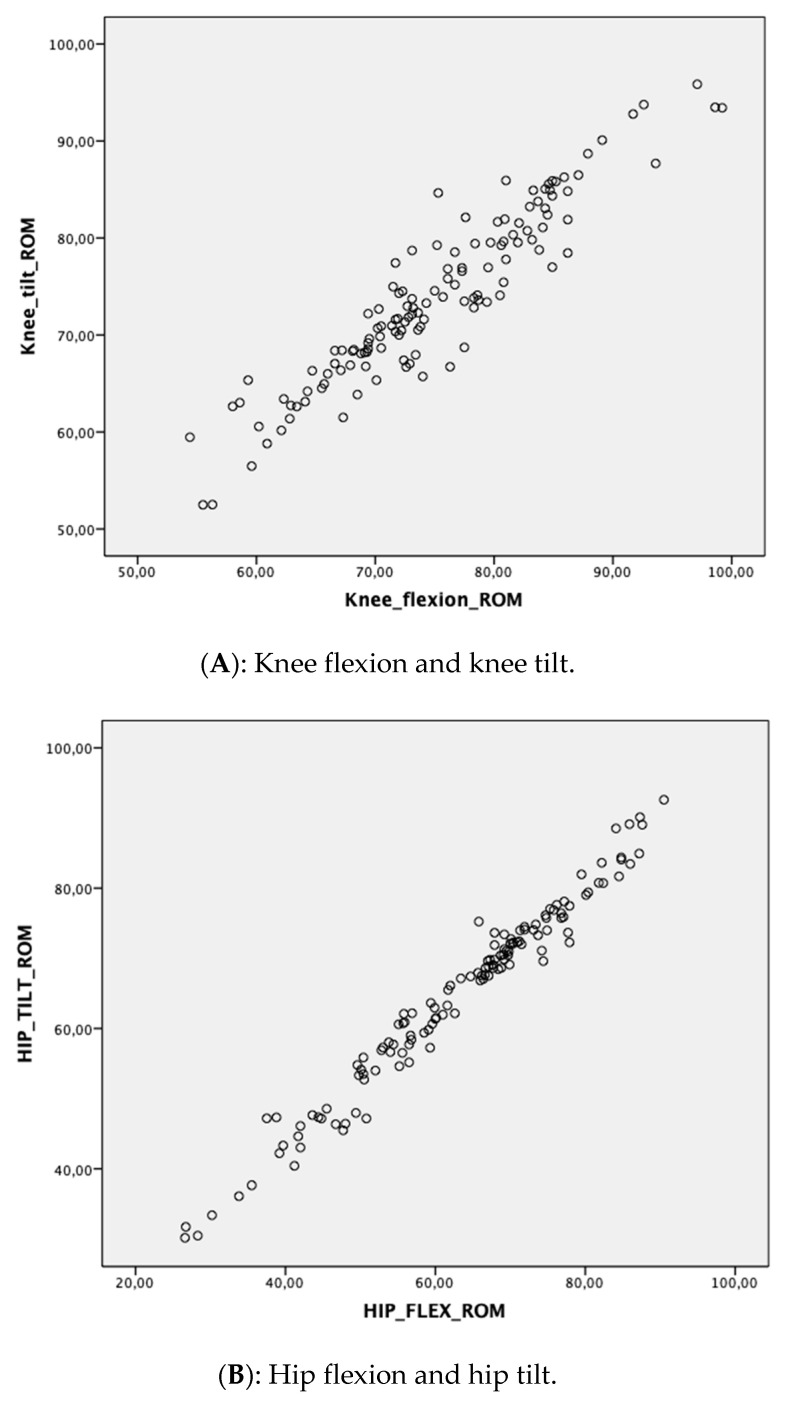

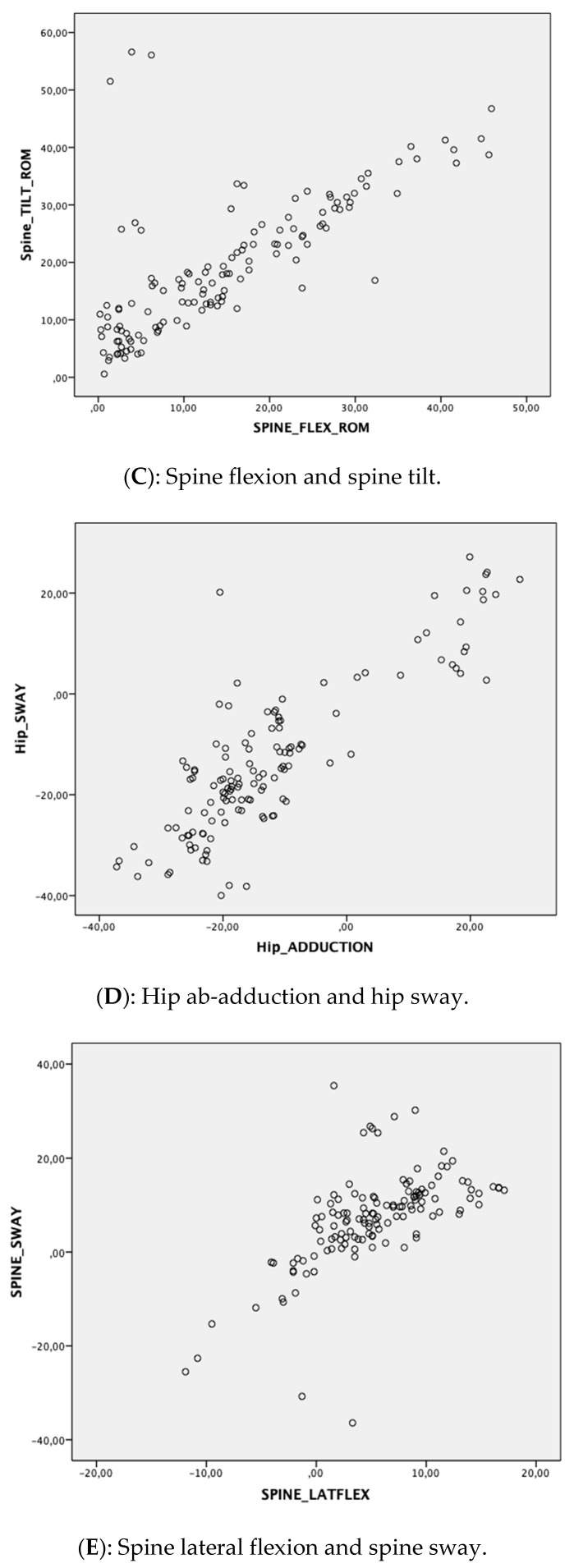
Correlation plots of movement directions tested. (Please note all decimals presented to be read as “.” instead of “,”).

All sagittal plane measurement correlations observed were very strong for the knee and hip (*r* = 0.929–0.988, *p* < 0.001). For sagittal plane spine assessment, the correlations were moderate (*r* = 0.708–0.728, *p* < 0.001). Frontal plane measurement correlations were moderate in size for the hip (ρ = 0.646–0.818, *p* < 0.001) and spine (ρ = 0.613–0.827, *p* < 0.001). Correlation plots of all measurement data combined (3 single leg squat trials) are presented in Figure 4.

## 4. Discussion

### 4.1. Main Findings

We studied the concurrent validity of a new 3D MoCap tool during a single leg squat task performed in a standing position. This validity was found to be good to excellent for all joint angles registered in the sagittal and frontal plane during three single leg squat trials. The highest correlations between systems were observed in the sagittal plane and were found to be most consistent. Hip and knee measurements performed best with the lowest difference between both systems.

### 4.2. Practical Relevance

The ability of the new MoCap tool to objectively measure sagittal and frontal plane joint angles is practically relevant: decreased spine, hip and knee flexion in single leg weight bearing and landing activities have been associated with an increased risk for the development of patellofemoral pain and anterior cruciate ligament knee (re)injury [5,7]. The new 3D MoCap tool can thus be applied in injury prevention screening and in progress evaluation after exercise interventions, aimed to optimize lower extremity kinematics. Parameters obtained are likely relevant to improve clinical outcomes. An important advantage of this new tool is that it can be applied on the pitch (on field) and in rehab and sports centre settings [19]. The kinematic data are then directly available in contrast with the more time consuming conventional motion capture systems [3,9,14].

Speed to be elicited in specific actions is one of the major goals in most type of sports. The capability of motion capture is unique in quantifying magnitude, timing and symmetry of segmental velocity. This may help physicians and physiotherapists to screen for deficits in any of those parameters and subsequently identify potential risk factors for future injury [42,43,44].

This study shows that easily accessible technology is likely to enter the market for a broad audience of professionals from different fields. The recent research interest from orthopedic and neurologic rehabilitation specialists in this type of technology shows that there is a loud call for quantification of many parameters in their specific patient populations [2,4,9,17,19,20,21,31,34,45]. Every movement related health problem would thus likely have its own clinical relevant movement parameters. Future studies will show what these parameters will be. Affordable new 3D MoCap systems will accelerate the pace of these developments. Larger datasets obtained at clinics as well as the pitch side are needed to further develop new knowledge.

### 4.3. Validity of Motion Capture Systems

Reliability of optimal tracking systems for gait analysis has been performed previously [16]. Optimal tracking systems show high reliability for pelvis, hip and knee ROM in the sagittal and frontal plane with mean precision errors less than 6°. Errors of 2° are considered acceptable, 2–5° reasonable and over 5° misleading. Markers attached to the skin move with respect to the underlying joints they intend to measure. This error is called soft tissue artefact (STA) and therefore invasive methods using radiostereometry are considered the gold standard in investigating joint motion because of its high accuracy [29]. A systematic review compared invasive methods against optimal tracking systems and found STA arising from tissue deformation, individual physical characteristics, marker location, type of segment and the nature of the performed tasks. The magnitude of STA measured was up to 40 mm at the thigh [46]. Also MoCap systems can be expected to be subject to STA, still any comparison with invasive methods seems to be lacking.

### 4.4. Other Studies

Recent studies investigating systems using inertial measurement units have found that these can distinguish between abnormal and sufficient performance on lower limb exercises with moderate to excellent accuracy. However, these studies assess movement patterns rather than specific joint angles, and use visual observation as reference standard rather than conventional 3D motion analysis [47,48]. Similar to this study, other markerless 3D MoCap tools such as the Kinect V2, have demonstrated stronger validity for sagittal plane kinematics than for the frontal plane during the single leg squat [45,49]. The difference in accuracy of sagittal plane versus frontal and transverse plane measurements can be explained from a biomechanical point of view. As sagittal plane movements are the largest in terms of ROM, a difference of some degrees between both methods will affect the distribution of outcomes relatively less than in frontal and transverse plane movements, which display a smaller ROM.

Additionally, differences between both MoCap tools were expected beforehand, because of the different manner in which joint angles are obtained. The conventional gold standard utilizes reflective markers, while the new tool employs active sensors. To calculate osteokinematic joint angles from the marker trajectories in 3D space, the conventional tool uses traditional Euler rotation sequences [37]. This method, however, suffers from gimbal lock and equations may be numerically unstable [38]. To express the orientation of a segment with respect to its primary segment (i.e., the joint angle) the new 3D MoCap tool uses quaternions that are derived directly from the sensors. This method overcomes the problem of gimbal lock, and extracting angles and axes of rotation is simpler and requires less computational steps [38].

In a comparable study, Zügner et al. found a measurement error of 2.8° for hip flexion/extension with a moderate correlation (ICC 0.75) which was for knee flexion/extension an error of 0.2° with a good correlation (ICC 0.83). Although these findings for the knee are comparable to our study, differences in hip findings could be due to the different sensor positions and performance tasks [17]. Another study compared IMU with OptiTrack measuring knee motion during a dynamic task as in our study [4]. They showed an error of measurement of 8° for flexion/extension (0.5° in our study) and poor correlation (Pearson’s R = 0.58 against our Pearson’s R = 0.94). This being lower may be the result of the dynamic jump task that influenced angle readings by eccentric gyroscopic fluctuations. Leardini et al. found small differences between an IMU and Vicon measures reporting a 5.0° error for knee flexion during a squat performance task which was between 0.5–1.5° in our study [18]. IMU and optical tracking systems both suffer from STA, yet most studies showed accepted measurement errors below the 5° bound. In our study, all hip and knee motion errors were lower than 2° and lower than 5° for spine measurements with good to excellent correlation.

### 4.5. Strengths and Limitations

To better reflect a clinical setting, squat depth was not controlled for in the testing protocol. It was previously reported that subjects are able to produce consistent sagittal plane range of motion without monitoring [35,36]. Our findings are in line with those as indeed we found consistent peak hip and knee flexion angles during the three trials of single leg squat. Spine flexion and lateral flexion however were less stable on repeated single leg squatting. This is acknowledged in the clinic where aberrant spine movements distract attention of the clinician. Although these spine positional changes are present hip and knee angles remained consistent.

The application studied shows adequate validity for MoCap in a clinical and on-site test setting. This type of assessment with easily accessible and low coast technology will likely offer new opportunities for clinicians as well as researchers to capture data that where prohibited for scientists in high laboratory setting.

The frequency of 50 Hz of the SportsLapp application is lower than the OptiTrack MoCap system. The validity data obtained can thus not be generalized to higher speed movements. The velocity threshold for adequate MoCap with this new system during higher speed movements should be subject of future study.

Questions can be raised regarding the placement of markers and sensors. Units placed on muscular areas, such as the lateral thigh, may have been subject to more artefacts due to muscle contraction and skin displacement than others, which were placed on bony landmarks. Marker placement on less or non-muscular areas like the lateral femoral condyle for the upper leg marker, may be considered to further refine the current protocol. A previous study comparing inertial sensors with an optoelectronic system reported lower errors on movement tracking when assessing a prosthesis when compared to a healthy human leg [50]. On top of this, artefacts may have occurred as a result of the inertial sensors were fixated with elastic tape over the cluster markers, prohibiting a rigid connection between the two.

## 5. Conclusions

This study shows that a new 3D MoCap tool utilizing active sensors has good to excellent concurrent validity for sagittal and frontal plane knee, hip and spine angle measurements assessed during a single leg squat task. Future studies are needed to investigate other variables like through range movement angles and velocity parameters.

## Figures and Tables

**Table 1:** Subject Characteristics.

	Total Group (*n* = 44)	Female (*n* = 20)	Male (*n* = 24)
Age (yrs)	22.8 (3.3) (18–30)	22.9 (3.6) (18–30)	22.7 (3.2) (18–30)
Height (cm)	175.3 (10.1) (155.0–197.0)	167 (5.7) (155–177)	182 (7.4) (170–197)
Weight (kg)	72.1 (11.6) (53.6–103.2)	64.1 (7.1) (53.6–82.2)	78.9 (10.6) (64.1–103.2)
Sport participation (hrs/wk)	5.4 (4.5) (1–25)	6.9 (5.2) (1–25)	3.1 (2.4) (1–8)
Activity level (Tegner score)	6.5 (1.3) (5–10)	5.8 (1.1) (3–7)	7.0 (1.2) (5–10)

Data are presented as mean (standard deviation) (range). Abbreviations: M = male; F = female; yrs = years; cm = centimetre; kg = kilogram; hrs = hours; wk = week.

**Table 2:** Correlations between measures with OptiTrack and SportsLapp motion capture systems for sagittal and frontal plane angles of knee, hip and spine.

**Trial 1**	Knee Flexion/Tilt *	Hip Flexion/Tilt *	**Hip Ab- Adduction/Sway**	Spine Flexion/Tilt *	**Spine Lateral Flexion/Sway**
OptiTrack (°)	73.8 (9.1) (71.1–76.6)	60.8 (14.7) (56.3–65.3)	−8.1 (17.1) (−13.3–−2.9)	13.8 (10.9) (10.5–17.1)	3.8 (5.7) (2.1–5.6)
SportsLapp (°)	73.3 (9.1) (70.6–76.1)	62.9 (13.4) (58.9–67.1)	−8.5 (18.3) (−13.9–−3.0)	18.1 (11.4) (14.6–21.6)	4.6 (10.2) (1.5–7.8)
Mean diff (°) (95%CI)	−0.5 (−1.5–0.5)	2.1 (1.3–2.9)	−0.4 (8.2) (–2.5–0.3)	−4.3 (−6.8–−1.17)	0.8 (8.1) (0.4–3.2)
Correlation	*r* = 0.936	*r* = 0.985	ρ = 0.818	*r* = 0.713	ρ = 0.827
**Trial 2**	Knee Flexion/Tilt *	Hip Flexion/Tilt *	**Hip Ab- Adduction/Sway**	Spine Flexion/Tilt *	**Spine Lateral Flexion/Sway**
OptiTrack (°)	75.0 (8.6) (72.4–77.6)	63.7 (14.7) (59.2–58.2)	−13.2 (14.1) (17.5–−8.9)	16.3 (12.1) (12.6–20.0)	6.3 (5.0) (4.7–7.8)
SportsLapp (°)	73.8 (8.6) (71.2–76.4)	64.9 (14.1) (60.6–69.2)	−14.5 (14.0) (−18.8–−10.3)	20.7 (11.9) (17.1–24.4)	7.4 (11.0) (4.0–10.7)
Mean diff (°) (95%CI)	−1.2 (−2.2–0.3)	1.2 (0.5–1.9)	−1.3 (−3.6–1.0)	−4.4 (−7.2–−1.6)	1.1 (−1.6–3.8)
Correlation	*r* = 0.929	*r* = 0.988	ρ = 0.704	*r* = 0.708	ρ = 0.641
**Trial 3**	Knee Flexion/Tilt *	Hip Flexion/Tilt *	**Hip Ab- Adduction/Sway**	Spine Flexion/Tilt *	**Spine Lateral Flexion/Sway**
OptiTrack (°)	76.0 (9.2) (73.1–78.8)	64.7 (13.6) (60.6–68.9)	−14.9 (13.1) (−18.9–−11.0)	14.7 (11.8) (11.1–18.3)	5.5 (5.3) (3.9–7.1)
SportsLapp (°)	74.6 (8.8) (71.9–77.3)	66.4 (13.0) (62.4–70.3)	−16.4 (12.5) (−20.2–−12.6)	19.8 (12.1) (16.1–23.4)	9.1 (9.4) (6.3–12.0)
Mean diff (°) (95%CI)	−1.4 (−2.3–−0.4)	1.7 (0.9–2.5)	−1.5 (−3.8–0.74)	−5.1 (−7.8–−2.4)	3.6 (1.3–5.9)
Correlation	*r* = 0.944	*r* = 0.982	ρ = 0.646	*r* = 0.728	ρ = 0.613

Data are presented in degrees as mean (standard deviation) and (95% confidence interval). Abbreviations: diff = difference; r = Pearson’s R correlation; ρ = Spearman’s Rho correlation. * = Normally distributed data.

## References

[1] Kivlan B.R. (2012). Functional Performance Testing of the Hip in Athletes. Int. J. Sports Phys. Ther..

[2] Rojas-Valverde D., Gómez-Carmona C.D., Gutiérrez-Vargas R., Pino-Ortega J. (2019). From big data mining to technical sport reports: The case of inertial measurement units. BMJ Open Sport Exerc. Med..

[3] Dingenen B., Malfait B., Vanrenterghem J., Verschueren S.M.P., Staes F.F. (2014). The reliability and validity of the measurement of lateral trunk motion in two-dimensional video analysis during unipodal functional screening tests in elite female athletes. Phys. Ther. Sport.

[4] Ajdaroski M., Tadakala R., Nichols L., Esquivel A. (2020). Validation of a device to measure knee joint angles for a dynamic movement. Sensors.

[5] Graci V., Van Dillen L.R., Salsich G.B. (2012). Gender differences in trunk, pelvis and lower limb kinematics during a single leg squat. Gait Posture.

[6] Hewett T.E., Torg J.S., Boden B.P. (2009). Video analysis of trunk and knee motion during non-contact anterior cruciate ligament injury in female athletes: Lateral trunk and knee abduction motion are combined components of the injury mechanism. Br. J. Sports Med..

[7] Pollard C.D., Sigward S.M., Powers C.M. (2010). Limited hip and knee flexion during landing is associated with increased frontal plane knee motion and moments. Clin. Biomech..

[8] McLean S.G., Walker K., Ford K.R., Myer G.D., Hewett T.E., Van Den Bogert A.J. (2005). Evaluation of a two dimensional analysis method as a screening and evaluation tool for anterior cruciate ligament injury. Br. J. Sports Med..

[9] Munro A., Herrington L., Carolan M. (2012). Reliability of 2-dimensional video assessment of frontal-plane dynamic knee valgus during common athletic screening tasks. J. Sport Rehabil..

[10] Munro A., Herrington L., Comfort P. (2017). The Relationship between 2-Dimensional Knee-Valgus Angles During Single-Leg Squat, Single-Leg-Land, and Drop-Jump Screening Tests. J. Sport Rehabil..

[11] Ekegren C.L., Miller W.C., Celebrin R.G., Eng J.J., MacIntyre D.L. (2009). Reliability and validity of observational risk screening in evaluating dynamic knee valgus. J. Orthop. Sports Phys. Ther..

[12] Stensrud S., Myklebust G., Kristianslund E., Bahr R., Krosshaug T. (2011). Correlation between two-dimensional video analysis and subjective assessment in evaluating knee control among elite female team handball players. Br. J. Sports Med..

[13] Willson J.D., Davis I.S. (2008). Utility of the frontal plane projection angle in females with patellofemoral pain. J. Orthop. Sports Phys. Ther..

[14] Willson J.D., Ireland M.L., Davis I. (2006). Core strenght and lower extremity alignment during single leg squats. Med. Sci. Sports Exerc..

[15] Stickler L., Finley M., Gulgin H. (2015). Relationship between hip and core strength and frontal plane alignment during a single leg squat. Phys. Ther. Sport.

[16] McGinley J.L., Baker R., Wolfe R., Morris M.E. (2009). The reliability of three-dimensional kinematic gait measurements: A systematic review. Gait Posture.

[17] Zügner R., Tranberg R., Timperley J., Hodgins D., Mohaddes M., Kärrholm J. (2019). Validation of inertial measurement units with optical tracking system in patients operated with Total hip arthroplasty. BMC Musculosketlet. Disord..

[18] Leardini A., Lullini G., Giannini S., Berti L., Ortolani M., Caravaggi P. (2014). Validation of the angular measurements of a new inertial-measurement-unit based rehabilitation system: Comparison with state-of-the-art gait analysis. J. NeuroEng. Rehabil..

[19] Islam R., Bennasar M., Nicholas K., Button K., Holland S., Mulholland P., Price B., Al-Amri M. (2020). A Nonproprietary Movement Analysis System (MoJoXlab) Based on Wearable Inertial Measurement Units Applicable to Healthy Participants and Those With Anterior Cruciate Ligament Reconstruction Across a Range of Complex Tasks: Validation Study. JMIR mHealth uHealth.

[20] Witchel H.J., Oberndorfer C., Needham R., Healy A., Westling C.E.I., Guppy J.H., Bush J., Barth J., Herberz C., Roggen D. (2018). Thigh-derived inertial sensor metrics to assess the sit-to-stand and stand-to-sit transitions in the timed up and go (TUG) Task for quantifying mobility impairment in multiple sclerosis. Front. Neurol..

[21] De Brabandere A., Emmerzaal J., Timmermans A., Jonkers I., Vanwanseele B., Davis J. (2020). A Machine Learning Approach to Estimate Hip and Knee Joint Loading Using a Mobile Phone-Embedded IMU. Front. Bioeng. Biotechnol..

[22] Bravi M., Gallotta E., Morrone M., Maselli M., Santacaterina F., Toglia R., Foti C., Sterzi S., Bressi F., Miccinilli S. (2020). Concurrent validity and inter trial reliability of a single inertial measurement unit for spatial-temporal gait parameter analysis in patients with recent total hip or total knee arthroplasty. Gait Posture.

[23] Teufl W., Miezal M., Taetz B., Fröhlich M., Bleser G. (2018). Validity, test-retest reliability and long-term stability of magnetometer free inertial sensor based 3D joint kinematics. Sensors.

[24] Tegner Y., Lysholm J. (1985). Rating systems in the evaluation of knee ligament injuries. Clin. Orthop. Relat. Res..

[25] World Medical Association (2013). World Medical Association Declaration of Helsinki: Ethical principles for medical research involving human subjects. JAMA.

[26] Central Committee on Research Involving Human Subjects. https://english.ccmo.nl/investigators/legal-framework-for-medical-scientific-research/laws/medical-research-involving-human-subjects-act-wmo.

[27] (1998). Medical Research Involving Human Subjects Act. Wet Medisch-Wetenschappelijk Onderzoek met Mensen.

[28] Tranberg R., Saari T., Zügner R., Kärrholm J. (2011). Simultaneous measurements of knee motion using an optical tracking system and radiostereometric analysis (RSA). Acta Orthop..

[29] Zügner R., Tranberg R., Lisovskaja V., Shareghi B., Kärrholm J. (2017). Validation of gait analysis with dynamic radiostereometric analysis (RSA) in patients operated with total hip arthroplasty. J. Orthop. Res..

[30] Thewlis D., Bishop C., Daniell N., Paul G. (2013). Next-generation low-cost motion capture systems can provide comparable spatial accuracy to high-end systems. J. Appl. Biomech..

[31] Carse B., Meadows B., Bowers R., Rowe P. (2013). Affordable clinical gait analysis: An assessment of the marker tracking accuracy of a new low-cost optical 3D motion analysis system. Physiotherapy.

[32] SportsLapp. http://www.sportslapp.com.

[33] Crossley K.M., Zhang W.J., Schache A.G., Bryant A., Cowan S.M. (2011). Performance on the single-leg squat task indicates hip abductor muscle function. Am. J. Sports Med..

[34] Nakagawa T.H., Moriya E.T.U., MacIel C.D., Serrão F.V. (2012). Trunk, pelvis, hip, and knee kinematics, hip strength, and gluteal muscle activation during a single-leg squat in males and females with and without patellofemoral pain syndrome. J. Orthop. Sports Phys. Ther..

[35] Whatman C., Hing W., Hume P. (2011). Kinematics during lower extremity functional screening tests—Are they reliable and related to jogging?. Phys. Ther. Sport.

[36] Zeller B.L., McCrory J.L., Ben Kibler W., Uhl T.L. (2003). Differences in kinematics and electromyographic activity between men and women during the single-legged squat. Am. J. Sports Med..

[37] Slabaugh G.G. Computing Euler Angles from a Rotation Matrix. http://gregslabaugh.name/publications/euler.pdf.

[38] Kuipers J.B., Mladenov I.M., Naber G.L. (2000). Quaternions and Rotation Sequences. Geometry, Integrability and Quantization.

[39] Masuda T., Ishida A., Cao L., Morita S. (2008). A proposal for a new definition of the axial rotation angle of the shoulder joint. J. Electromyogr. Kinesiol..

[40] Dijkstra F., den Besten G., Factic B.V. (2018). 3Dynamics Angles Definitions for Measuring and Presenting 3D Motions of Human Joints.

[41] Chan Y.H. (2005). Biostatistics 104. Corrleational analysis. Sing. Med. J..

[42] Morishige Y., Harato K., Kobayashi S., Niki Y., Matsumoto M., Nakamura M., Nagura T. (2019). Difference in leg asymmetry between female collegiate athletes and recreational athletes during drop vertical jump. J. Orthop. Surg. Res..

[43] Ithurburn M.P., Paterno M.V., Thomas S., Pennell M.L., Evans K.D., Magnussen R.A., Schmitt L.C. (2019). Change in Drop-Landing Mechanics Over 2 Years in Young Athletes After Anterior Cruciate Ligament Reconstruction. Am. J. Sports Med..

[44] King E., Richter C., Franklyn-Miller A., Wadey R., Moran R., Strike S. (2019). Back to Normal Symmetry? Biomechanical Variables Remain More Asymmetrical Than Normal During Jump and Change-of-Direction Testing 9 Months After Anterior Cruciate Ligament Reconstruction. Am. J. Sports Med..

[45] Eltoukhy M., Kelly A., Kim C.Y., Jun H.P., Campbell R., Kuenze C. (2016). Validation of the Microsoft Kinect^®^ camera system for measurement of lower extremity jump landing and squatting kinematics. Sports Biomech..

[46] Peters A., Galna B., Sangeux M., Morris M., Baker R. (2010). Quantification of soft tissue artifact in lower limb human motion analysis: A systematic review. Gait Posture.

[47] Kianifar R., Lee A., Raina S., Kulic D. Classification of squat quality with inertial measurement units in the single leg squat mobility test. Proceedings of the Annual International Conference of the IEEE Engineering in Medicine and Biology Society, EMBS.

[48] Whelan D.F., O’Reilly M.A., Ward T.E., Delahunt E., Caulfield B. (2017). Technology in rehabilitation: Evaluating the single leg squat exercise with wearable inertial measurement units. Methods Inf. Med..

[49] Mentiplay B.F., Hasanki K., Perraton L.G., Pua Y.H., Charlton P.C., Clark R.A. (2018). Three-dimensional assessment of squats and drop jumps using the Microsoft Xbox One Kinect: Reliability and validity. J. Sports Sci..

[50] Seel T., Raisch J., Schauer T. (2014). IMU-based joint angle measurement for gait analysis. Sensors.

